# Self-Catalyzed Growth and Characterization of In(As)P Nanowires on InP(111)B Using Metal-Organic Chemical Vapor Deposition

**DOI:** 10.1186/s11671-016-1427-4

**Published:** 2016-04-19

**Authors:** Jeung Hun Park, Marta Pozuelo, Bunga P. D. Setiawan, Choong-Heui Chung

**Affiliations:** Department of Materials Science and Engineering, University of California Los Angeles, Los Angeles, California 90095 USA; Department of Materials Science and Engineering, Hanbat National University, Daejeon, 305-719 Republic of Korea; Present address: IBM T.J. Watson Research Center, Yorktown Heights, New York, 10598 USA

**Keywords:** Self-catalyst, In(As,P) nanowires, Vapor-liquid-solid process, Metal-organic chemical vapor deposition, Raman spectroscopy, Scanning transmission electron microscopy, X-ray photoelectron spectroscopy

## Abstract

We report the growth of vertical <111>-oriented InAs_*x*_P_1−*x*_ (0.11 ≤ *x* ≤ 0.27) nanowires via metal-organic chemical vapor deposition in the presence of indium droplets as catalysts on InP(111)B substrates at 375 °C. Trimethylindium, tertiarybutylphosphine, and tertiarybutylarsine are used as the precursors, corresponding to P/In and As/In molar ratios of 29 and 0.01, respectively. The as-grown nanowire growth morphologies, crystallinity, composition, and optical characteristics are determined using a combination of scanning and transmission electron microscopies, electron diffraction, and X-ray photoelectron, energy dispersive X-ray, and Raman spectroscopies. We find that the InAs_*x*_P_1−*x*_ nanowires are tapered with narrow tops, wider bases, and In-rich In-As alloy tips, characteristic of vapor-liquid-solid process. The wires exhibit a mixture of zinc blende and wurtzite crystal structures and a high density of structural defects such as stacking faults and twins. Our results suggest that the incorporation of As into InP wires decreases with increasing substrate temperature. The Raman spectra obtained from the In(As)P nanowires reveal a red-shift and lower intensity of longitudinal optical mode relative to both InP nanowires and InP(111)B bulk, due to the incorporation of As into the InP matrix.

**Electronic supplementary material:**

The online version of this article (doi:10.1186/s11671-016-1427-4) contains supplementary material, which is available to authorized users.

## Background

One-dimensional semiconducting nanowires have attracted considerable attention for potential applications in electronics, optoelectronics, and chemical and biological sensing devices [1–4]. Over the past two decades, considerable efforts have been devoted to the synthesis of elemental (group IV [5, 6]), binary compound (groups III–V [7, 8], groups II–VI [9, 10]), and metal oxide nanowires [11]. The growth of compound nanowires is desirable for high-frequency electronics and optoelectronics, such as light-emitting devices operating at high frequency [12, 13], high electron drift velocity devices [14], and photodiodes [15, 16] due to their low energy band gaps and very high electron mobility. In typical vapor-liquid-solid (VLS) growth [17] of groups III–V nanowires, gold is the most commonly used catalyst material but is undesirable because gold forms deep level traps [18]. To overcome this problem, previous studies have shown that nanowires of groups III–V binary compounds can been grown via VLS process using group III metal as the catalyst [19–25]. However, relatively few studies have reported catalyst-free growth of ternary compound nanowires [26–29].

In this letter, we report the self-catalyzed growth of In(As)P alloy nanowires on InP(111) substrate by metal-organic chemical vapor deposition (MOCVD). Using liquid indium as the catalyst, we optimized the growth conditions to obtain vertically oriented In(As)P nanowires on InP(111)B substrate. The morphology and crystallinity of In(As)P nanowires are examined using scanning- and transmission- electron microscopies (SEM and TEM). The composition and optical properties of as-grown nanowires are determined using energy-dispersive spectroscopy (EDS) in the TEM, X-ray photoelectron spectroscopy (XPS), and micro-Raman spectroscopy.

## Methods

All of our nanowire growth experiments are carried out in a Veeco D125 MOCVD reactor using trimethylindium (TMIn), tertiarybutylarsine (TBA), and tertiarybutylphosphine (TBP) as indium (In), arsine (As), and phosphine (P) precursors, respectively. Details of the MOCVD system and the experimental procedure used to grow nanowires can be found in refs. [21, 30–32]. Briefly, single-crystalline InP(111)B wafers (0.5° miscut and 5 × 10^6^ Ω-cm resistivity) are used as substrates. The samples are first placed in the reactor and annealed at 550 °C in 2.6 × 10^−3^ mol/s of H_2_ for 360 s. The H_2_ flow is then switched off, and TBP was introduced for 900 s at 7.4 × 10^−6^ mol/s. After annealing, the temperature was lowered to 375 °C. Liquid indium droplets are deposited at 375 °C by feeding 8.4 × 10^−7^ mol/s of TMIn for 12 s. In(As)P nanowires are grown by feeding TMIn, TBP, and TBA into the reactor at the flow rates of 5.0 × 10^−5^ mol/s, 7.4 × 10^−6^ mol/s, and 8.3 × 10^−8^ mol/s, respectively. These flow rates correspond to P/In and As/In molar ratios of 29 and 0.01, respectively. The total pressure is always kept constant at 60 Torr during the entire growth process. The growth is terminated after 300 s by switching off the precursors and cooling the samples to room temperature at ~0.65 °C/s by flowing H_2_ for 540 s. Note that the absolute temperatures in our MOCVD reactor are accurate to within 25 K, and the substrate temperature is found to decrease monotonically from the center to the edge of the substrate by ~40 K [30]. All of the results presented here, except those in Figs. 2a and 3a, were obtained from the nanowires extracted from the center of the substrate, where the temperature is ~375 °C.

Morphologies of the as-grown samples are determined using FEI Nova 600 field emission SEM operated at 10 kV accelerating voltage. Individual nanostructures, which are mechanically exfoliated from the growth wafer and transferred into Cu TEM grid, are further characterized using TEM, scanning TEM (STEM), selected area electron diffraction (SAED), and EDS (Oxford Instruments) in an FEI Titan STEM operated at 300 kV. The SEM and TEM images are processed using ImageJ [33], TEM Imaging and Analysis (TIA), and Digital Micrograph as a means to measure the sizes and shapes of the nanowires. Crystal structure and crystallinity of the nanowires are determined from the TEM and SAED data. Nanowire composition is obtained using EDS point and/or line scans acquired from more than ten nanowires and XPS (Omicron XPS/UPS system) survey scans and core-level spectroscopy. The XPS measurements are performed on mechanically exfoliated In(As)P nanowires transferred onto 200-nm-thick Au coated Si(111) substrate using Al K_α_ (1486.6 eV) as the excitation source with normal (90°) incidence of the beam with respect to substrate surface. The expected resolution in our XPS measurements is <1 at.%. The Au 4f_7/2_ (84.1 eV) [34] and the adventitious C 1s (284.5 eV) [35] peak positions are used as the references to correct for any charging induced shifts in the XPS data. Core-level spectroscopic data were deconvoluted [36] using a mixture of Gaussian and Lorentzian curves after Shirley background subtraction [34]. From the fitted curves and accounting for the atomic sensitivity factors, elemental compositions within the nanowires are determined [37, 38]. Raman spectroscopic measurements are performed using an optical fiber-coupled Raman microscope system (Jovin Yvon-Horiba LabRam HR-800) on an Olympus BX41 optical microscope. The Raman detector is equipped with a liquid nitrogen cooling system and a motorized stage. Raman signals are obtained in a backscattering configuration using He-Cd laser (325 nm) and ×100 ultraviolet objective lens. The laser spot size is approximately 1 μm. The nominal resolution of the measured spectra is within <0.5 cm^−1^. Substrate-heating-induced spectral shift was excluded by keeping the excitation power (on the substrate) below 0.5 mW. The Raman system was calibrated using a reference Si(111) substrate. Ensembles of mechanically exfoliated InP or In(As)P nanowires on InP(111)B substrate are used to identify the incorporation of As into InP matrix. Least squares minimization model with the Voigt profile was employed to fit the obtained spectra from semiconducting materials InP and In(As)P [39].

## Results and Discussion

**Fig. 1:**
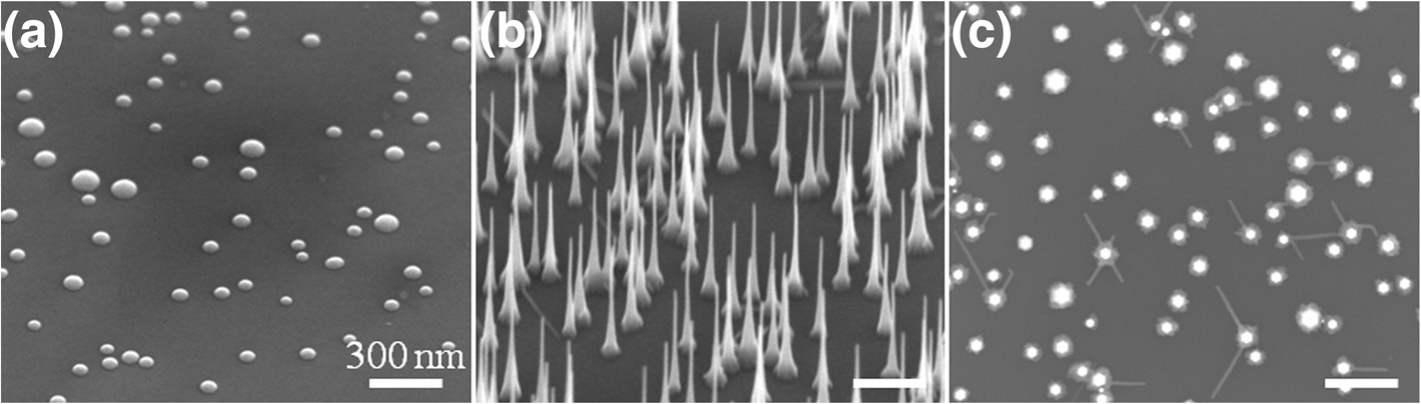
Scanning electron microscopy (SEM) images showing (**a**) a 45° tilted view of In droplets deposited at 375 °C on InP(111)B by feeding 8.4 × 10^−7^ mol/s of TMIn for 12 s, (**b**) 45° tilted, and (**c**) top view of In(As)P nanowires on InP(111)B grown at 375 °C over 300 s by feeding TMIn, TBP, and TBA into the reactor at the flow rates of 5.0 × 10^−5^, 7.4 × 10^−6^, and 8.3 × 10^−8^ mol/s, respectively

Figure 1a is a typical SEM image acquired from an InP(111)B sample after In deposition at 375 °C. The image shows a distribution of dome-shaped In mounds with an average size of 61 ± 14 nm and an areal density of (2 ± 0.2) × 10^9^ cm^−2^. At 375 °C, we expect that the as-deposited In forms liquid droplets, which solidify upon cooling, to yield the observed shapes. Figure 1b shows In(As)P nanowires grown on InP(111)B substrate. The nanowires exhibit hexagonal cross-sectional shapes (see Fig. 1c) and are tapered with tip and base diameters of 9 ± 3 nm and 42 ± 14 nm, respectively. The average length of nanowires is found to be 579 ± 15 nm, which corresponds to a vertical growth rate of 2.4 ± 0.2 nm/s. The uncertainties in the nanowire diameters and lengths are determined from the corresponding measurements over 50 different nanowires. The areal density of nanowires is ~(2.5 ± 0.2) × 10^9^ cm^−2^, nearly the same as that of the In droplets. That is, each of the In droplets gives rise to one nanowire. However, the base diameters of the nanowires are consistently lower than that of the In droplets situated on the substrate prior to nanowire growth; this is expected and is due to a change in the contact angle between the liquid droplet and solid surface underneath that occurs during growth [40, 41]. These observations are consistent with our expectation that In droplets act as catalysts for the growth of nanowires via VLS process [17]. All of the above results are similar to those reported for the self-catalyzed VLS growth of InP and InPSb nanowires [21, 31, 32].

**Fig. 2:**
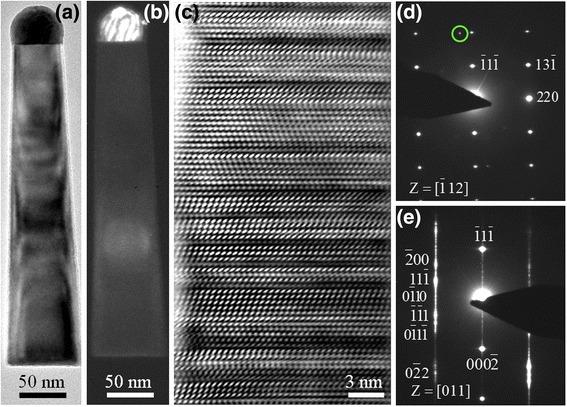
**a** Bright field, **b** dark field, and **c** Fourier-filtered high-resolution transmission electron microscopy (TEM) images of an In(As)P nanowire grown on InP(111)B nanowire using the same conditions as in Fig. 1. The image in **b** is obtained using the diffraction spot highlighted by a *green circle* in (**d**). This particular nanowire was extracted from a region on the substrate (likely at a lower temperature) that is different from the region shown in Fig. 1. **d**, **e** Selected area electron diffraction (SAED) patterns obtained from different wires oriented to different zone axes $\left[\overline{1}12\right]$ and $\left[011\right]$, respectively. An additional diffraction spot seen inside the *green circle* in **d** is due to crystalline In. **e** SAED of the whole nanowire shown in **c** indexed as zinc blende in [011] zone axis. Additional spots are indexed as wurtzite crystal structure in $\left[2\overline{1}\overline{1}0\right]$ zone axis, which is parallel to $\left[011\right]$. The high-resolution TEM image in **c** was Fourier-filtered using ImageJ package to reduce the background noise

Figure 2a shows a representative bright-field TEM image of a single In(As)P nanowire. (This particular nanowire is collected from the edge of InP(111)B substrate, where the substrate temperature may have been lower than at the center during deposition [30].) We see the presence of a smooth, nearly hemispherical dome-shaped feature with darker contrast at the tip of the wire. The nanowire is tapered with a diameter of 45 nm at the tip, a diameter of 70 nm at the base, and a length of 335 nm. The resulting morphology of the nanowire, grown along the substrate edges, is that it is less tapered than that in Fig. 1b. We found that the nanowires grown at the center (edge) of the substrate have higher (lower) aspect ratios with narrower (wider) bases. These observations are qualitatively similar to the previously reported morphologies of self-catalyzed InP [21] and InPSb [30] nanowires. We speculate that this observed variation in nanowire morphologies across the growth substrate is a consequence of temperature-dependent rates of migration and/or evaporation of indium away from the wire tips [21]. Figure 2b is a dark-field TEM image obtained using the (112) diffraction spot associated with tetragonal In [42], highlighted by a green circle in the SAED pattern (see Fig. 2d); under these particular imaging conditions, the wire tip appears brighter in contrast, indicative of the presence of crystalline In or In-rich alloy phase in the tip. Figure 2c is a high-resolution TEM image of an individual In(As)P nanowire showing a high density of twins and stacking faults. Figure 2d, e shows SAED patterns of the nanowires shown in Fig. 2a, c, respectively. From the SAED data, we assign the crystal structure of the nanowire in Fig. 2a, c as zinc blende with $\left[\overline{1}12\right]$ and [011] zone axes, respectively. Additional spots are indexed as wurtzite crystal structure in $\left[2\overline{1}\overline{1}0\right]$ zone axis, which is parallel to [011]. The streaks along [111] are indicative of the structural defects oriented perpendicular to this direction. (These results are consistent with the expectation that non-nitride groups III–V compounds crystallize in the zinc blende crystal structure [43]. Within the In(As)P alloy crystal, In atoms reside in group III sub-lattice positions while As and P atoms randomly occupy group V sub-lattice positions [43].) The lattice parameter of the wire shown in Fig. 2a as measured from the diffraction pattern in Fig. 2d is 5.94 ± 0.02 Å. Assuming that Vegard’s law [43] is valid for InP-InAs system and using the lattice constants of zinc blende structured InP (5.87 Å) [29] and InAs (6.06 Å) [44, 45], we estimate that the incorporated content of As in the InAs_*x*_P_1−*x*_ nanowire is approximately *x* = 0.36 ± 0.10. Following a similar approach, we analyzed the SAED pattern in Fig. 2e and estimate an As content *x* ~ 0.27 for the nanowire shown in Fig. 2c.

**Fig. 3:**
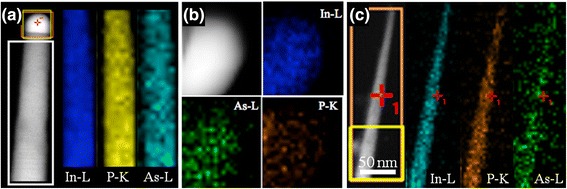
**a** Scanning transmission electron microscopy (STEM) image of the same In(As)P nanowire shown in Fig. 2 and two-dimensional energy dispersive spectroscopy (EDS) elemental maps obtained from the region enclosed by a *white rectangle*. **b** EDS elemental maps from the wire tip highlighted by a *red square* in (**a**). **c** STEM image and EDS elemental maps of In(As)P nanowire acquired from the center of the substrate

Figure 3a, b shows representative STEM images and EDS data of In(As)P nanowires and the wire tips, respectively, collected from the edge region of the substrate. (For comparison, Fig. 3c shows a representative STEM image and EDS data of In(As)P nanowires grown at the center region of the substrate.) Two-dimensional (2D) EDS maps, color coded for clarity, in Fig. 3a show spatial distributions of In, P, and As along the length of the nanowire. We find that the elemental composition, within the measurement uncertainties, is uniform throughout the wire. The average As content within the wires grown at the substrate edges is 17.4 ± 8.7 at.%, while the As content in those wires grown at the substrate center is 11.6 ± 6.4 at.%. Figure 3b shows the EDS data obtained from the wire tip, highlighted by a square in the STEM image in Fig. 3a. The spectral maps reveal the presence of P and As in addition to In inside the wire tip. However, the exact composition of the wire tip could not be determined due to the limited resolution in these measurements. Due to the limited solubility of P and As (in both liquid at the growth temperature, 375 °C, and solid at room-temperature phases of In [46], In-P [47], and In-As [48]) and the diffraction spot in Fig. 2d (enclosed by a green circle) corresponding to tetragonal-structured In (or In-rich alloy), we expect that the concentrations of P and As in the wire tip are negligible [49]. Similar results have been reported for In-catalyzed growth of InP and InPSb nanowires [31, 32]. Based on the identification of In-rich phase in the wire tip from the SAED and EDS data, we suggest that the growth of InAsP nanowires occurred via VLS process with liquid In-rich In-As alloy droplets as catalysts.

**Fig. 4:**
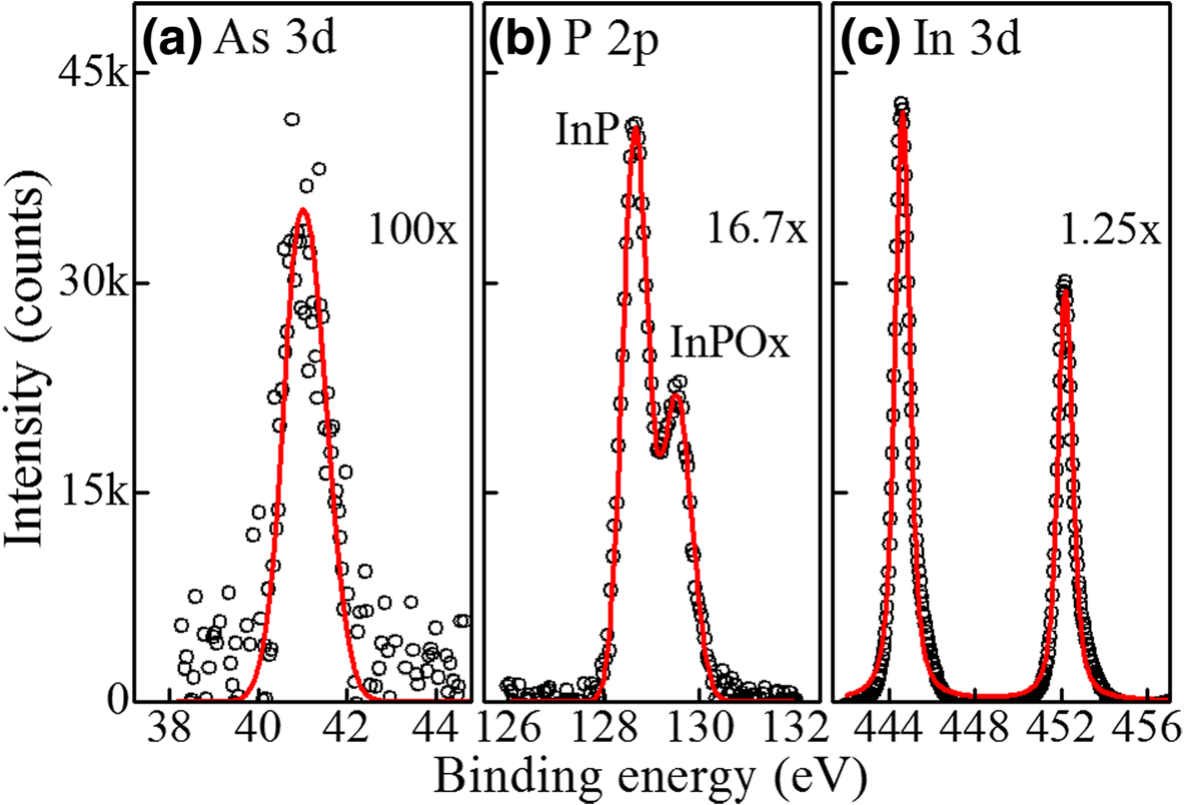
X-ray core-level spectra of single In(As)P nanowire. **a** As 3d. **b** P 2p. **c** In 3d. The *red curves* are the least squares fits to the data obtained using a mixture of Gaussian and Lorentzian curves after Shirley background subtraction

Figure 4 shows core-level X-ray photoelectron spectroscopic data obtained from the mechanically exfoliated In(As)P nanowires. The measured XPS data were least-square fitted using the Voigt profile. The core-level spectra show peaks at binding energies of 444.1 eV for In 3d_5/2_ [50] and 451.4 eV for In 3d_3/2_ [50]. The presence of P is indicated by the observation of two 2p core-level peaks, one at 128.6 eV corresponding to P from InP and the other at 129.5 eV corresponding to oxidation due to air exposure [50]. The presence of As 3d leads to a peak at 41.7 eV, corresponding to As 3d_5/2_ [50]. From the XPS data, taking into account the atomic sensitivity factors (0.677 for As 3d, 4.539 for In 3d_5/2_, and 0.486 for P 2p) [38, 50], we calculated the As content from the deconvoluted spectra to be 10.7 ± 0.8 at.%. The resulting compositions of the ensemble of nanowires are InAs_0.11_P_0.89_, which is consistent with the STEM-EDS analysis of the individual In(As)P nanowire in Fig. 3c. All of the above data clearly indicate that the incorporated contents of As into the wire increase with the decrease in the substrate temperature. The substrate-temperature-dependent variation in InAs_*x*_P_1−*x*_ nanowire composition could arise due to differences in the rates of P and As atom incorporation into the nanowire, high surface diffusivity of As adatoms toward InP substrate [51], or surface migration of In catalyst from the wire tip at higher substrate temperatures (~375 °C) [52]. Our observation is consistent with the previous studies of InAsP [53] and GaAsP [54] systems.

**Fig. 5:**
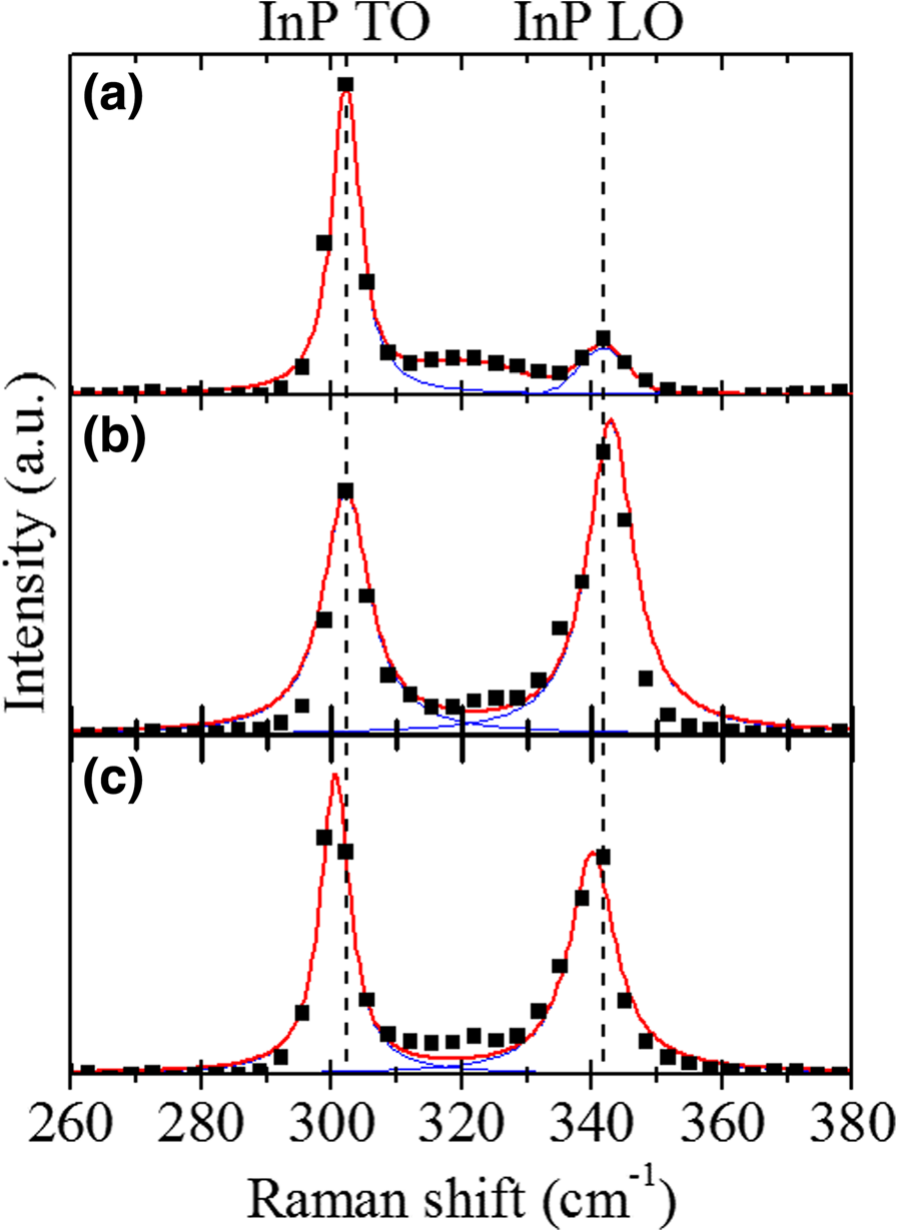
Raman spectra obtained from **a** InP(111)B wafer, **b** InP nanowires on InP(111)B wafer, and (**c**) In(As)P nanowires on InP(111)B wafer. The *red* and *blue curves* correspond to the least squares fits to the entire spectra and to the deconvoluted phonon modes using the Voigt profiles, respectively. InP TO and LO peaks are indicated with the *dashed lines*

Figure 5 shows the Raman spectrum obtained from In(As)P nanowires. The result is compared with Raman spectra of InP(111)B bulk wafer and InP nanowires (see Additional file 1: Figure S1) grown on InP(111)B substrate using In as the catalysts. Two peaks marked with the dashed lines are clearly visible that correspond to transverse optical (TO) and longitudinal optical (LO) phonons. For InP (111)B wafer, the TO and LO peaks are observed at 302.2 and 341.7 cm^−1^, respectively. For In(As)P nanowire, the TO and LO peaks are located at 300.5 and 340 cm^−1^, respectively. The intensities of both LO and TO peaks in the In(As)P nanowire sample (or InP nanowire) are higher than those in the InP(111)B wafer due to the selection rule and crystal anisotropy of low dimensional nanostructures [55]. (Both LO and TO are observed from ZB (111) surface but TO is only observed from ZB (110) surface.) However, the intensity of LO phonons in In(As)P nanowires is lower than that in InP nanowires. We attribute this result to larger atomic radius of arsenic compared to phosphorus and the expansion of lattice of In(As)P [29]. Moreover, the TO and LO peaks of In(As)P nanowires are relatively red-shifted toward the lower wave numbers by 1.5 and 2.8 cm^−1^, respectively, with the respect to InP nanowire. The observations in the reduction of LO peak intensity and the red-shift in In(As)P nanowires are attributed to the incorporation of more localized carriers (arsenic) in InP matrix and increased free carrier concentrations which lowers the width of the surface depletion layer in the nanowire [28, 56].

## Conclusions

We demonstrate the growth of vertical In-catalyzed In(As)P nanowires on InP(111)B substrate using metal-organic chemical vapor deposition. With the optimized growth parameters (the growth temperature at 375 °C, P/In molar ratio ~29 and As/In molar ratio ~0.01), we could obtain <111>-oriented InAs_*x*_P_1−*x*_ (0.11 ≤ *x* ≤ 0.27) nanowires via self-catalyzed VLS growth process. The nanowires exhibit a mixture of zinc blende and wurtzite crystal structures with a high density of structural defects. Interestingly, we observe that the content of As into InP wires is found to decrease with increasing substrate temperature. In addition, the red-shift and weak resonance of LO mode in In(As)P nanowires further support the incorporation of As into InP matrix. We expect that our experimental results could help engineer the physical properties of groups III–V nanowires.

## Additional file

Additional file 1: Figure S1. 45° tilted SEM image of InP nanowires on InP(111)B substrate grown at 365 °C for 300 s using TMIn (5.0 × 10^−5^ mol/s) and TBP (7.4 × 10^−6^ mol/s) precursors with the molar ratio of V/III = 29. Mechanically exfoliated InP nanowires are used as the reference for Raman spectroscopy measurements. (PNG 215 kb)

## Abbreviations

As: arsine
EDS: energy-dispersive spectroscopy
In: indium
LO: longitudinal optical phonon
MOCVD: metal-organic chemical vapor deposition
P: phosphine
SAED: selective area diffraction pattern
SEM: scanning electron microscope
STEM: scanning transmission electron microscope
TBA: tertiarybutylarsine
TBP: tertiarybutylphosphine
TEM: transmission electron microscope
TMIn: trimethylindium
TO: transverse optical phonon
XPS: X-ray photoelectron spectroscopy
