# Supplementary figures and images for: Self-Catalyzed Growth and Characterization of In(As)P Nanowires on InP(111)B Using Metal-Organic Chemical Vapor Deposition
Source: Nanoscale Res Lett. 2016 Apr 19;11:208. doi: 10.1186/s11671-016-1427-4 (PMC4837196; doi:10.1186/s11671-016-1427-4)

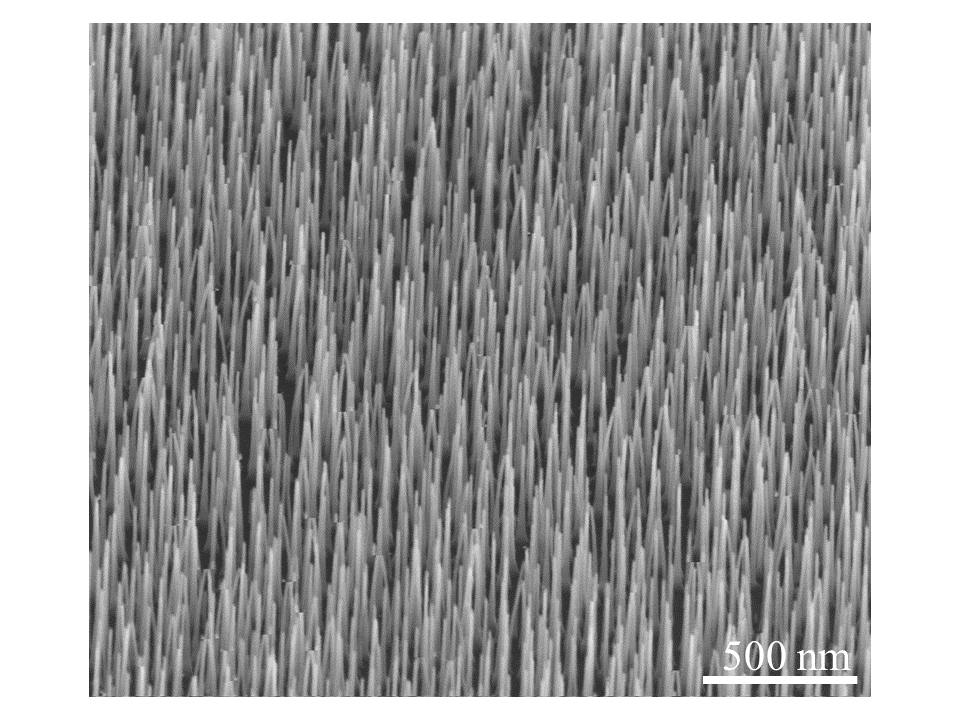

Supplement: Additional file 1: Figure S1. — 45° tilted SEM image of InP nanowires on InP(111)B substrate grown at 365 °C for 300 s using TMIn (5.0 × 10^−5^ mol/s) and TBP (7.4 × 10^−6^ mol/s) precursors with the molar ratio of V/III = 29. Mechanically exfoliated InP nanowires are used as the reference for Raman spectroscopy measurements. (PNG 215 kb) [file 11671_2016_1427_MOESM1_ESM.png]
